
JOURNAL INFORMATION
==============================
NLM Title Abbreviation: Biospektrum (Heidelb)
Journal ID: Biospektrum (Heidelb)
NLM Journal ID: 9615685

Biospektrum

ISSN: 0947-0867
EISSN: 1868-6249

ARTICLE INFORMATION
==============================
PMCID: PMC8501375
PMID: 34658539
DOI: 10.1007/s12268-021-1659-4
Article ID: 1659
Article version: 1
Subjects: Biotechnologie

Synthetische Glykobiotechnologie

Rexer Thomas rexer@mpi-magdeburg.mpg.de http://www.mpi-magdeburg.mpg.de/3461919/synthetic-glycobiotechnology

Son Tuan Hoang
Ruhnau Johannes
Reichl Udo
grid.419517.f 0000 0004 0491 802X Abteilung Bioprozesstechnik (BPT), Max-Planck-Institut für Dynamik komplexer technischer Systeme, Sandtorstraße 1, D-39106 Magdeburg, Deutschland
Electronic publication date: 2021 Oct 9
Print publication date: 2021
Volume: 27
Issue: 6
First page: 657
Last page: 659
Copyright: © Die Autoren 2021
License: Dieser Artikel wird unter der Creative Commons Namensnennung 4.0 International Lizenz veröffentlicht, welche die Nutzung, Vervielfältigung, Bearbeitung, Verbreitung und Wiedergabe in jeglichem Medium und Format erlaubt, sofern Sie den/die ursprünglichen Autor(en) und die Quelle ordnungsgemäß nennen, einen Link zur Creative Commons Lizenz beifügen und angeben, ob Änderungen vorgenommen wurden. Die in diesem Artikel enthaltenen Bilder und sonstiges Drittmaterial unterliegen ebenfalls der genannten Creative Commons Lizenz, sofern sich aus der Abbildungslegende nichts anderes ergibt. Sofern das betreffende Material nicht unter der genannten Creative Commons Lizenz steht und die betreffende Handlung nicht nach gesetzlichen Vorschriften erlaubt ist, ist für die oben aufgeführten Weiterverwendungen des Materials die Einwilligung des jeweiligen Rechteinhabers einzuholen. Weitere Details zur Lizenz entnehmen Sie bitte der Lizenzinformation auf http://creativecommons.org/licenses/by/4.0/deed.de.
License URL: https://creativecommons.org/licenses/by/4.0/

issue-copyright-statement: © The Author(s), under exclusive licence to Springer-Verlag GmbH Deutschland, ein Teil von Springer Nature 2021

==============================
The field of synthetic glycobiotechnology encompasses the synthesis and modification of free carbohydrates and carbohydrates linked to biomolecules. Our group develops bio-catalytic processes for the synthesis of carbohydrate building blocks, so-called sugar nucleotides, and cell-free multi-enzyme cascades to tailor carbohydrates linked to proteins. The technology can eventually help to advance our understanding of the roles of specific carbohydrates in nutrition and medicine and contribute to human health and well-being.

■ Zuckerstrukturen, in denen mehrere Monosaccharide über eine glykosidische Bindung verbunden sind, die Glykane, sind zentral für die Gesundheit des Menschen. Glykane kommen sowohl als freie Strukturen, z. B. in menschlicher Muttermilch, als auch kovalent an Biomoleküle gebunden vor. Die Erforschung der Funktionen und die Möglichkeit, diese zu synthetisieren oder zu verändern, führen zunehmend zu biotechnologischen Anwendungen. So wurde im Jahr 2013 mit Obinutuzumab erstmalig ein monoklonaler Antikörper für die Anwendung am Menschen zugelassen, bei dem die Zuckerstrukturen mittels gentechnischer Methoden gezielt modifiziert wurden [1]. Dieser weist im Vergleich zum nicht modifizierten Antikörper eine erhöhte Wirksamkeit bei komorbiden Patienten mit chronisch lymphatischer Leukämie auf. Des Weiteren erfolgt seit kurzem die Zugabe eines biotechnologisch hergestellten humanen Milchzuckers zu Säuglingsnahrungsprodukten. Die positive Auswirkung humaner Milchzucker auf die Entwicklung von Säuglingen wurde zuvor in zahlreichen Studien belegt [2]. Damit ist das Potenzial der Glykobiotechnologie aber noch lange nicht ausgeschöpft. Allein in der humanen Muttermilch befinden sich bis zu 200 verschiedene humane Milchzuckerstrukturen. Für die Konzeption und Durchführung weiterer Wirksamkeitsstudien, sowohl von Milchzuckern als auch von therapeutischen Proteinen mit unterschiedlichen Glykanstrukturen, wird eine Vielzahl dieser Substanzen benötigt. Insbesondere für die Produktion von Kleinstmengen hat sich die biokatalytische Synthese, d. h. die Nutzung von Kaskaden rekombinanter Enzyme als vielversprechend erwiesen [3]. Eine große Anzahl der zum Aufbau der Zuckerstrukturen benötigten Enzyme, die Glykosyltransferasen, können sehr effizient mittels Genexpression in *Escherichia coli* hergestellt werden [4]. Herausforderungen für die Etablierung von Plattformtechnologien zur Synthese und Modifizierung der Zuckerstrukturen sind insbesondere die Verfügbarkeit von günstigen Ausgangssubstraten sowie die Entwicklung von skalierbaren, robusten Syntheseverfahren.

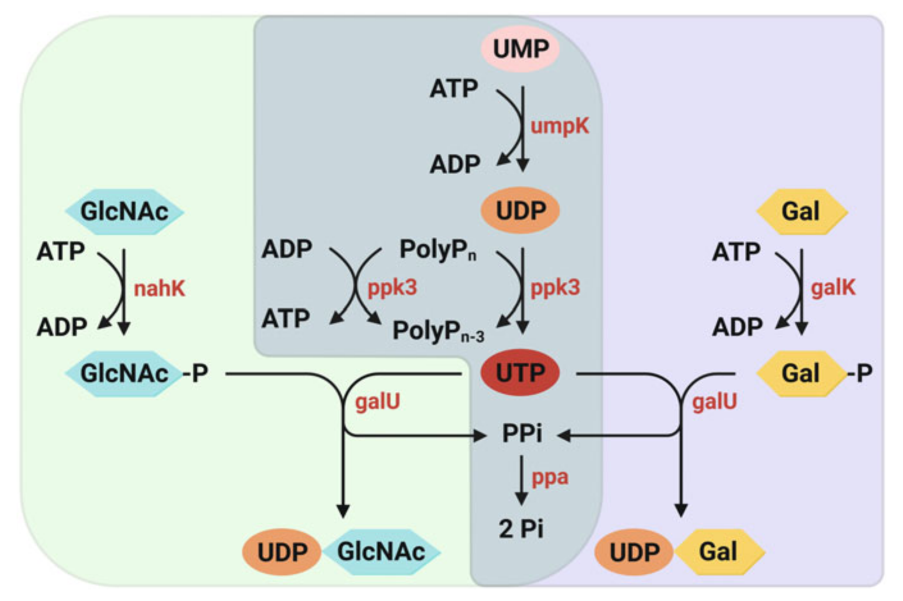

▲ **Abb. 1:** Enzymatisches Reaktionsnetzwerk zur Synthese von UDP-Gal und UDP-GlcNAc im Eintopfverfahren. Der Prozess ermöglicht die Synthese beider Nukleotidzucker aus den günstigen und in großen Mengen verfügbaren Ausgangssubstraten Polyphopshate (PolyP$_n$), Uridinmonophosphat (UMP), *N*-Acetylglucosamin (GlcNAc) bzw. Galactose (Gal). Adenosintriphosphat (ATP) wird zur Kostenersparnis *in situ* aus Adenosindiphosphat (ADP) und PolyP$_n$ regeneriert. Weitere Zwischenprodukte sind Uridindiphosphat (UDP), Uridintriphosphat (UTP), Pyrophosphat (PPi) und *N*-Acetylglucosamin-1-phosphat (GlcNAc-P) bzw. Galactose-1-phosphat (Gal-P). Als Endprodukt entsteht zudem Phosphat. Erstellt mit Biorender.com.

## Nukleotidzucker

Für die enzymatische Synthese von humanen Milchzuckern und Glykanen werden meistens Nukleotidzucker als Substrate benötigt. Die Nukleotidzucker nehmen dabei als Zuckerdonoren an der Reaktion teil, d. h. ein aktivierter Zuckerbaustein wird auf die vorhandene Zuckerstruktur übertragen. Das Problem: Die wichtigsten Nukleotidzucker sind kommerziell typischerweise nur in äußerst geringen Mengen verfügbar – zu

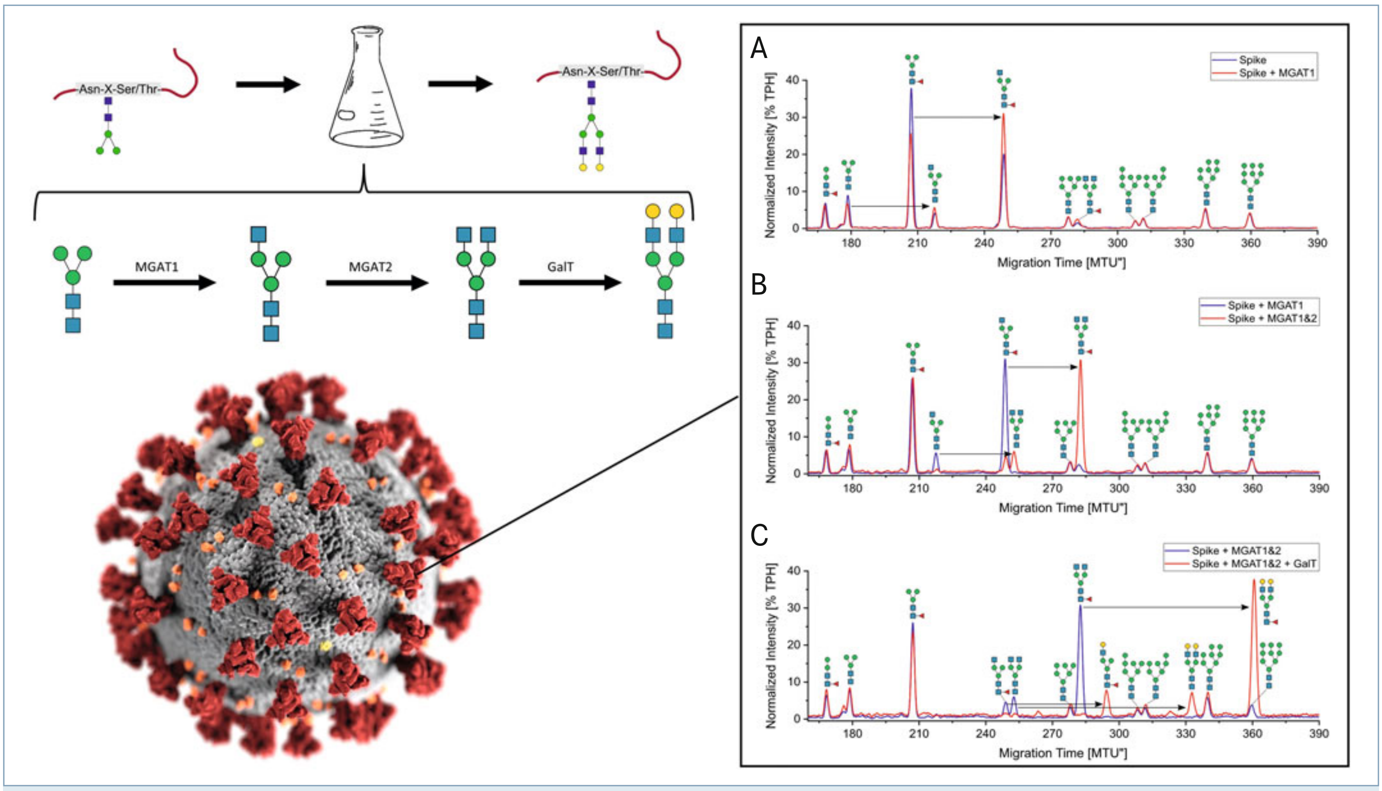

▲ **Abb. 2:** Enzymatische Veränderung der Glykane des rekombinanten SARS-CoV-2-Spike-Proteins zur Erforschung der Immunantwort unterschiedlicher Glykanhüllen. Hier wurde aus einfachen mannosylierten Strukturen komplexe Glykane mit GlcNAc und terminaler Galactose erzeugt. Rechts: Elektropherogramm des unprozessierten und prozessierten Spike-Proteins. **A**, Glykanhülle nach einer Reaktionszeit von 12 Stunden mittels der Glykosyltransferase MGAT1. **B**, 12 Stunden nach zusätzlicher Zugabe der MGAT2. **C**, 12 Stunden nach zusätzlicher Zugabe der GalT [7]. Glykansymbole: grün = Mannose; blau = GlcNAc; rot = Fucose; gelb = Galactose.

Preisen zwischen rund 2.000 und 15.000 Euro pro Gramm. In den vergangenen Jahren hat unser Team deshalb Reaktionskaskaden bestehend aus rekombinanten Enzymen zur Synthese von Nukleotidzuckern in Eintopfverfahren entwickelt [5]. Mittels zweier dieser Kaskaden können die Nukleotidzucker Uridindiphosphat-Galactose (UDP-Gal) und Uridindiphosphat-*N*-Acetylglucosamin (UDP-GlcNAc) synthetisiert werden. Diese Nukleotidzucker stellen neben Fucose und Sialinsäure bei enzymatischen Glykosylierungsreaktionen die Bausteine Galactose und GlcNAc als zentrale Bausteine von humanen Milchzuckern zum Transfer bereit (**Abb. 1**). Beide Kaskaden bestehen aus je sechs Enzymen. Als Ausgangssubstrate dienen die günstigen und in großen Mengen verfügbaren Chemikalien Polyphopshat, Uridinmonophosphat und *N*-Acetylglucosamin bzw. Galactose.

## Glykane

Rekombinante Glykosyltransferasen können auch zur gezielten Veränderung von Glykanen an Proteinen angewandt werden. Diese als *in vitro glycoengineering* bezeichnete Methode hat den Vorteil, vollkommen unabhängig vom Herstellprozess des Zielproteins durchführbar zu sein. Des Weiteren können mittels dieser Methode eine Reihe von Strukturen flexibel mit großer Homogenität generiert werden. Neben der Anwendung der Technologie auf monoklonale Antikörper und andere Proteine, könnte eine Anwendung insbesondere auch für die Modifikation viraler Proteine von Interesse sein. Diese sind wesentliche Bestandteile der Virusoberfläche und stellen meist das wichtigste Antigen zur Herstellung von Impfstoffen dar. Ein Beispiel ist das Spike-Protein des SARS-CoV-2-Erregers, welches eine essenzielle Rolle beim Eindringen der Viren in Wirtszellen spielt. Spike-Proteine weisen, wie die Oberflächenproteine vieler andere Viren, eine Glykanhülle auf. Diese Glykanhülle bestimmt mit, ob und wie das Immunsystem des Menschen auf Viren reagiert und ob eine Erkrankung hervorgerufen wird. Welchen Einfluss einzelne Glykanstrukturen von viralen Proteinen auf die Immunantwort haben, ist bisher jedoch noch wenig erforscht [6].

Virale Proteine können häufig rekombinant in Insektenzellsystemen produziert werden, u. a. auch zur Verwendung als Impfstoff. Um die Glykanstruktur dieser Proteine gezielt anzupassen, haben wir eine Kaskade bestehend aus drei rekombinanten Glykosyltransferasen, den GlcNAc-Transferasen MGAT1 und MGAT2 sowie der Galactosyltransferase GalT, etabliert. Unter Anwendung dieser Kaskade konnte die Glykanhülle des Coronavirus-Spike-Proteins von einer insektentypischen Glykanhülle in menschenähnliche Glykanstrukturen umgewandelt werden (**Abb. 2**, [7]). In Zusammenarbeit mit der Arbeitsgruppe von Prof. Dr. Dunja Bruder, AG Infektionsimmunologie am Institut für Medizinische Mikrobiologie und Krankenhaushygiene Magdeburg der Universität Magdeburg, soll zukünftig der Einfluss verschiedener modifizierter Varianten auf die Immunantwort im Mausmodell untersucht werden.

## *In vitro*-Transfer von Glykanen auf Peptide

Die Verlinkung von Glykanen und Proteinen zur Synthese von Glykoproteinen findet bisher fast ausschließlich innerhalb tierischer Zellen statt. Bakterielle Produktionssysteme wie *E. coli* weisen im Allgemeinen keinen

Glykosylierungsapparat auf und eignen sich daher nicht zur rekombinanten Produktion von Glykoproteinen. Mehrere Forschungsgruppen arbeiten jedoch an Ansätzen, um Peptide und später vielleicht auch Proteine nachträglich, d. h. zellfrei im Reagenzglas, zu glykosylieren. Wichtig bei der Etablierung dieser Prozesse ist, dass die Glykane nur an vorgegebene, bestimmte Sequenzen am Protein übertragen werden können. Dafür stehen mittlerweile komplexe rekombinante Proteine zur Verfügung [8]. Ein weiterer Schlüssel zu diesem Prozess ist die Verfügbarkeit von lipidverlinkten Glykanen, denn nur von diesen kann das Glykan auf die entsprechende Sequenz am Zielprotein übertragen werden. Der enzymatische Aufbau dieser lipidgebundenen Vorstufen durch eine lineare Kaskade von rekombinanten Glykosyltransferasen konnte bereits erfolgreich entwickelt werden [9, 10]. Das langfristige Ziel dieser Technologie ist es, die volle Kontrolle über die Glykosylierung von Biomolekülen zu erlangen und damit das Potenzial der synthetischen Glykobiotechnologie voll auszuschöpfen.


Danksagung

Dr. Thomas Rexer dankt der DFG für die finanzielle Förderung (Projektnummer: 458633485). Des Weiteren danken die Autoren Frau Prof. Dr. Dunja Bruder, AG Infektionsimmunologie am Institut für Medizinische Mikrobiologie und Krankenhaushygiene Magdeburg, Otto-von-Guericke-Universität Magdeburg, für die gute Zusammenarbeit im Rahmen des gemeinsamen Forschungsprojektes.

Funding note

Open Access funding enabled and organized by Projekt DEAL.

Das Forschungsteam „Synthetische Glykobiotechnologie“ am Max-Planck-Institut Magdeburg im September 2021. Von links: Tuan Hoang Son, Johannes Ruhnau, Sebastian Kleeberg, Alberto Alcala, Annabell Darr, Dr. Mariana Juarez, Dr. Thomas Rexer. Es fehlen: Lisa Wenzel und Anja Bastian. Foto: Frania Jaqueline Zuniga.


Literatur

[1] Walsh G Biopharmaceutical benchmarks 2018 Nat Biotechnol 2018 36 1136 1145 10.1038/nbt.4305 30520869
[2] Bode L Human milk oligosaccharides: every baby needs a sugar mama Glycobiology 2012 22 1147 1162 10.1093/glycob/cws074 22513036 PMC3406618
[3] Bych K Miks MH Johanson T Production of HMOs using microbial hosts — from cell engineering to large scale production Curr Opin Biotechnol 2019 56 130 137 10.1016/j.copbio.2018.11.003 30502637
[4] Rexer TFT Laaf D Gottschalk J Enzymatic synthesis of glycans and glycoconjugates Adv Biochem Eng Biotechnol 2021 175 231 280 33052414 10.1007/10_2020_148
[5] Mahour R Marichal-Gallardo PA Rexer TFT Multi-enzyme cascades for the in vitro synthesis of guanosine diphosphate L-fucose ChemCatChem 2021 13 1981 1989 10.1002/cctc.202001854
[6] Schön K Lepenies B Goyette-Desjardins G Impact of protein glycosylation on the design of viral vaccines Adv Biochem Eng Biotechnol 2021 175 319 354 32935143 10.1007/10_2020_132
[7] Ruhnau J Grote V Juarez-Osorio M Cell-free glycoengineering of the recombinant SARS-CoV-2 spike glycoprotein Front Bioeng Biotechnol 2021 9 699025 10.3389/fbioe.2021.699025 34485255 PMC8415157
[8] Ramírez AS Boilevin J Biswas R Characterization of the single-subunit oligosaccharyltransferase STT3A from Trypanosoma brucei using synthetic peptides and lipid-linked oligosaccharide analogs Glycobiology 2017 27 525 535 10.1093/glycob/cwx017 28204532 PMC5421464
[9] Rexer TFT Schildbach A Klapproth J One pot synthesis of GDP-mannose by a multi-enzyme cascade for enzymatic assembly of lipid-linked oligosaccharides Biotechnol Bioeng 2018 115 192 205 10.1002/bit.26454 28922469 PMC5765510
[10] Rexer TFT Wenzel L Hoffmann M Synthesis of lipid-linked oligosaccharides by a compartmentalized multi-enzyme cascade for the in vitro N-glycosylation of peptides J Biotechnol 2020 322 54 65 10.1016/j.jbiotec.2020.07.003 32653637
